# Genome-Wide Insights into *Streptomyces* Novel Species Qhu-G9 and Its Potential for Enhancing Salt Tolerance and Growth in *Avena sativa* L. and *Onobrychis viciifolia* Scop

**DOI:** 10.3390/plants14142135

**Published:** 2025-07-10

**Authors:** Xin Xiang, Xiaolan Ma, Hengxia Yin, Liang Chen, Jiao Li, Wenjing Li, Shuhan Zhang, Chenghang Sun, Benyin Zhang

**Affiliations:** 1 College of Eco-Environmental Engineering, Qinghai University, Xining 810016, China; 2023970035@qhu.edu.cn (X.X.); ys220713000143@qhu.edu.cn (X.M.); ys240860010130@qhu.edu.cn (L.C.); ys230713000135@qhu.edu.cn (J.L.); ys230713000152@qhu.edu.cn (W.L.); 230710024139@qhu.edu.cn (S.Z.); 2 State Key Laboratory of Plateau Ecology and Agriculture, Qinghai University, Xining 810016, China; 3 Institute of Medicinal Biotechnology, Chinese Academy of Medical Sciences & Peking Union Medical College, Beijing 100050, China; sunchenghang@imb.pumc.edu.cn

**Keywords:** *Streptomyces*, plant growth-promoting rhizobacteria, salt stress, plant growth promotion, antioxidant enzymes, biochemical responses

## Abstract

With the increasing severity of global climate change and soil salinization, the development of microorganisms that enhance crop salt tolerance has become a critical focus of agricultural research. In this study, we explored the potential of a novel *Streptomyces* species Qhu-G9 as a plant growth-promoting rhizobacterium (PGPR) under salt stress conditions, employing whole-genome sequencing and functional annotation. The genomic analysis revealed that Qhu-G9 harbors various genes related to plant growth promotion, including those involved in phosphate solubilization, indole-3-acetic acid (IAA) biosynthesis, antioxidant activity, and nitrogen fixation. A total of 8528 coding genes were annotated in Qhu-G9, with a significant proportion related to cell metabolism, catalytic activity, and membrane transport, suggesting its broad growth-promoting potential. In vitro experiments demonstrated that Qhu-G9 exhibited strong iron siderophore production, IAA synthesis, phosphate solubilization, and 1-aminocyclopropane-1-carboxylate (ACC) deaminase activity, all of which correlate with its plant growth-promoting capacity. Further plant growth trials revealed that Qhu-G9 significantly enhances the growth of *Avena sativa* and *Onobrychis viciifolia* seedlings under salt stress conditions, improving key physiological parameters, such as chlorophyll content, relative water content, and photosynthetic efficiency. Under salt stress conditions, inoculation with Qhu-G9 resulted in notable increases in total biomass, root length, and plant height. Biochemical analyses further confirmed that Qhu-G9 alleviates the oxidative damage induced by salt stress by boosting antioxidant enzyme activities, reducing peroxide levels, and promoting the accumulation of osmotic regulators. These findings suggest that Qhu-G9 holds great promise as a PGPR that not only promotes plant growth, but also enhances plant tolerance to salt stress; thus, it has significant agricultural potential.

## 1. Introduction

Soil salinization is a phenomenon that results in the deterioration of soil characteristics and a decline in soil quality, leading to reduced crop yields. This, in turn, places a significant constraint on agricultural productivity and the sustainable development of both resources and the environment [1]. It is projected that, by 2050, half of the global cultivated land will be affected by salinization, while 20–30% of irrigated agricultural lands will experience yield reductions due to salt accumulation, thereby presenting a formidable challenge to global food security [2]. Current methods for the reclamation of saline–alkaline soils primarily include the establishment of drainage and irrigation systems, deep plowing, the cultivation of salt-tolerant plants, the application of biodegradable organic fertilizers, and the addition of chemical agents [3,4]. However, while these approaches may offer short-term relief from salinity stress, they are often associated with high costs and the potential for secondary pollution, making it difficult to achieve the desired outcomes in salinized soil management. In this context, the use of salt-tolerant microbial inoculants to improve soil quality and enhance plant resistance to salinity represents a promising and significant direction for research in the amelioration of soil salinization.

The interactions between soil microorganisms and plant root systems give rise to unique microenvironments, wherein resident bacteria, fungi, and other microbes form stable community structures through cooperation and competition [5]. These soil microorganisms play a pivotal role in regulating the rhizosphere environment, modulating plant growth and development, as well as enhancing system productivity [6]. Growing evidence has demonstrated that the application of microbial inoculants can effectively mitigate the detrimental effects of salinity on plant seedlings in salinized soils [7,8,9]. Among these, plant growth-promoting rhizobacteria (PGPR) are of primary significance. These bacteria not only contribute ions and regulate the ion balance in the soil, thereby enhancing soil fertility, but also improve the rhizosphere environment and salt tolerance of plants [10,11]. Furthermore, they provide an array of essential nutrients that are readily absorbed and utilized by plants, facilitating the restoration of saline–alkaline lands through vegetative rehabilitation [12]. For instance, the use of salt-tolerant PGPR promotes soil microbial metabolism and—through processes such as phosphorus solubilization, potassium release, nitrogen fixation, iron chelation, and the synthesis of plant hormones—fosters plant growth [13,14]. Therefore, research on the application of salt-tolerant PGPR for the reclamation and utilization of saline–alkaline soils holds significant promise.

Soil salinization often results in poor root development, premature senescence, reduced crop quality, and increased susceptibility to diseases [15]. The application of PGPR offers a novel, economically viable, ecologically sound, and sustainable approach to protecting plants from salt stress and enhancing agricultural productivity [16]. PGPR promote plant growth through direct or indirect mechanisms, effectively mitigating the damage caused by salt stress to crops [12]. These beneficial effects are often linked to their plant growth-promoting properties, such as phosphorus solubilization, potassium release, nitrogen fixation, iron chelation, indole-3-acetic acid (IAA) synthesis, and exopolysaccharide (EPS) production [17]. Li et al. demonstrated that salt-tolerant PGPRs capable of synthesizing IAA stimulate the plant’s endogenous IAA production, indirectly increasing proline levels and antioxidant enzyme activities and, thereby, enhancing salt tolerance and improving crop yield [18]. Qurashi et al. observed that PGPRs producing EPSs on chickpea seed surfaces form biofilms that retain moisture, significantly increasing seed germination rates [19]. EPSs can also interact with Na^+^ and K^+^ ions in saline soils to reduce salt ion accumulation, while facilitating nutrient adsorption and improving soil aggregation, permeability, and aeration [20]. Consequently, research on salt-tolerant PGPRs, which alleviate salt stress and promote plant growth, has gained considerable attention in recent years [21,22,23]. This research has primarily focused on genera such as *Bacillus*, *Pseudomonas*, *Oligotrophus*, *Serratia*, *Azospirillum*, and *Rhizobium* [24,25]. However, studies on actinomycetes, particularly *Streptomyces*, as PGPR in relation to plant growth promotion and salt tolerance remain limited.

*Streptomyces* is the largest genus within the phylum Actinobacteria and is widely distributed in soils, with over 900 species reported to date [26]. Many *Streptomyces* strains exhibit the ability to solubilize inorganic and organic phosphates, produce siderophores, and secrete 1-aminocyclopropane-1-carboxylate (ACC) deaminase [27]. Some *Streptomyces* species have been reported to alleviate salt stress in plants. For example, *S. lasalocidi* JCM 3373 promotes soybean root development and salt stress-responsive gene expression by secreting indole-3-aldehyde [28]. Similarly, the salt-tolerant endophytic *Streptomyces* sp. KLBMP5084 enhances tomato salt tolerance by modulating physiological, biochemical, and gene expression pathways related to salt stress resistance [29]. Therefore, *Streptomyces* holds significant potential for application in protecting plants from abiotic stresses and in improving soil quality.

*Avena sativa* L. and *Onobrychis viciifolia* Scop. are well-suited forage crops for cultivation in the arid and semi-arid regions of northwestern China, exhibiting moderate tolerance to salinity [30,31,32]. *O. viciifolia*, in particular, is rich in protein, amino acids, and a variety of essential minerals, and it is also noted for its high palatability [32]. The cultivation of these forage species not only enhances soil quality and boosts agricultural productivity, but it also helps to mitigate competition between forage and food crops. However, the widespread salinization of soils imposes significant constraints on the growth and productivity of *A. sativa* and *O. viciifolia*. Although approaches such as genetic improvement and molecular breeding have shown promise in enhancing the salt stress resilience of these crops, they are often time-consuming and resource-intensive [33]. This underscores the urgent need for the development of innovative and efficient strategies to improve their salt tolerance and overall stress resistance. In a preliminary study, a *Streptomyces* strain, Qhu-G9, isolated from a drought-prone habitat in Qinghai Province, China, was identified as a potential new species using polyphasic taxonomy methods, and it was named *S. haixigobicum* Qhu-G9 [34]. However, its plant growth-promoting and salt tolerance properties remain unclear. In this study, we performed a whole-genome analysis and plant growth-promoting trait evaluation of the Qhu-G9 strain, and we then assessed its effect on plant growth and salt tolerance in potted *A. sativa* and *O. viciifolia* under salt stress conditions. The results of this study provide valuable insights for the development of novel salt-tolerant microbial agents and their application in the efficient cultivation of crops in saline–alkaline soils.

## 2. Results

### 2.1. Phylogenetic Relationships and Comparative Genomics of Qhu-G9

**Figure 1:**
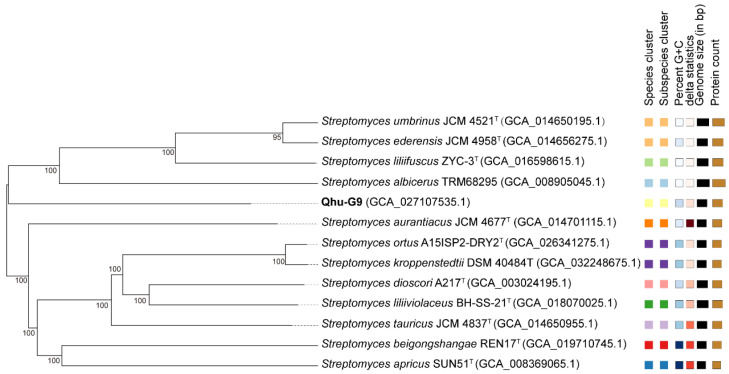
Phylogenetic analysis based on the Genome BLAST Distance Phylogeny (GBDP) distances derived from the genome sequence of Qhu-G9. Branch lengths represent genomic distances calculated using the GBDP distance formula *d*5. Numerical values above branches indicate GBDP pseudo-bootstrap support based on 100 replicates (shown only when exceeding 60%). The tree is midpoint-rooted. Type strains are indicated with a superscript “T”. From left to right, the figure sequentially displays the following: species clusters (denoted by different colors indicating distinct species); subspecies clusters (color-coded by subspecies); genomic G+C content; total genome size; and the proportion of protein-coding genes.

Building on previous whole-genome sequencing [34], a phylogenetic tree was constructed using the Genome BLAST Distance Phylogeny (GBDP) method to analyze the genomic relationships of the Qhu-G9 strain with closely related strains (Figure 1).

The results reveal that Qhu-G9 forms a distinct phylogenetic branch. This finding was further corroborated by a phylogenetic tree based on 81 conserved core genes, which was generated using the Up-to-Date Bacterial Core Gene version 2 (UBCG2) software (Figure S1). Together, these analyses reinforce the taxonomic placement of Qhu-G9 as a novel species within the genus *Streptomyces*, which is in agreement with the identification obtained through the previous polyphasic taxonomic approaches assessed in this study [34].

**Figure 2:**
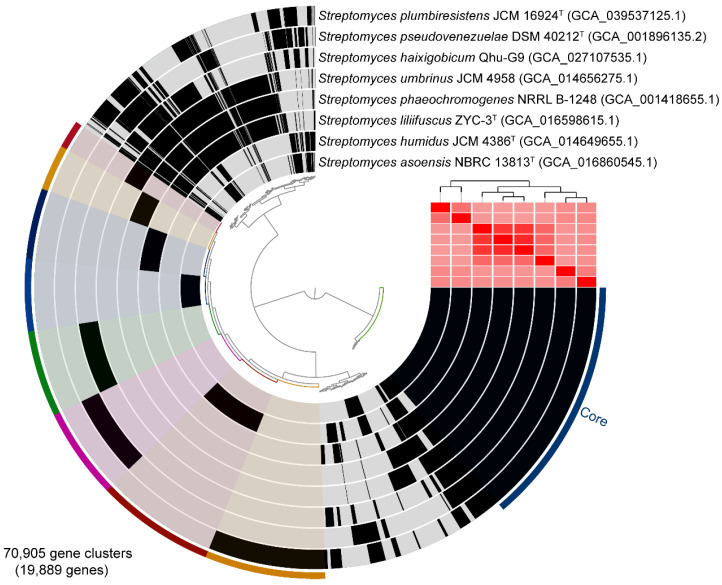
Whole-genome comparative analysis of Qhu-G9 and its closely related strains. The outermost ring represents the unique genomic clusters, color-coded for differentiation, of each strain. The seven colors, ranging from red to orange, represent the unique gene clusters corresponding to the genomes of *S. umbrinus* JCM 4958; *S. phaeochromogenes* NRRL B-1248; *S. liliifuscus* ZYC-3^T^; *S. asoensis* NBRC 13813^T^; Qhu-G9; *S. pseudovenezuelae* DSM 40212^T^; *S. humidus* JCM 4386^T^; and *S. plumbiresistens* JCM 16924^T^. “Core” indicates the gene clusters that are shared by all these species. The following rings display the geometric homogeneity index and functional homogeneity index. In the heatmap, deeper shades of red indicate higher ANIb (ANI algorithm using BLAST) similarity.

The genomic comparison of Qhu-G9 and the closely related strains was performed using the anvi’o v8.0 tool (Figure 2). A total of 70,905 gene clusters and 19,889 genes were identified, among which 33,298 core gene clusters were common across the strains. Notably, Qhu-G9 harbors 1341 unique gene clusters (Table S1), which are predominantly associated with metabolic functions, such as “Carbohydrate transport and metabolism”, “Amino acid transport and metabolism”, “Lipid transport and metabolism”, “Mobilome: prophages, transposons”, “Replication, recombination, and repair”, and “Energy production and conversion”. These findings suggest that the unique genomic content of Qhu-G9 may play a significant role in its adaptation to the extreme environmental conditions of the Tibetan Plateau.

Additionally, a search of the Qhu-G9 genome using Blast v2.9.0 revealed the presence of 1319 virulence-associated genes (Figure S2 and Table S2), which were primarily related to Colibactin, FbpABC, Capsule, Type III Secretion System (T3SS), and pyoverdine. Comparative genomic island prediction conducted with IslandPath-DIMOB and SIGI-HMM tools identified a total of 1026 genomic islands within the Qhu-G9 genome (Figure S3 and Table S3). Among these, 804 genomic islands lacked functional annotations. Of the remaining annotated islands, approximately one-quarter encode mobile genetic elements (specifically transposases of insertion sequences (IS), which can insert into coding regions through various transposition mechanisms, leading to gene mutations, deletions, and inversions). Alternatively, insertion into upstream regions can affect the expression of downstream genes. These mobile genetic elements likely contribute to the Qhu-G9′s ability to adapt to extreme environmental fluctuations, facilitating survival under harsh conditions.

### 2.2. Functional Annotation of the Qhu-G9 Genome

Based on the predicted gene sequences, functional annotations were conducted by aligning the genome against various databases, including COG, GO, KEGG, Pfam, Swiss-Prot, TrEMBL, and Nr. A total of 8525 protein-coding genes were annotated (Table S4), and the gene distributions across each database are summarized in Table 1.

**Figure 3:**
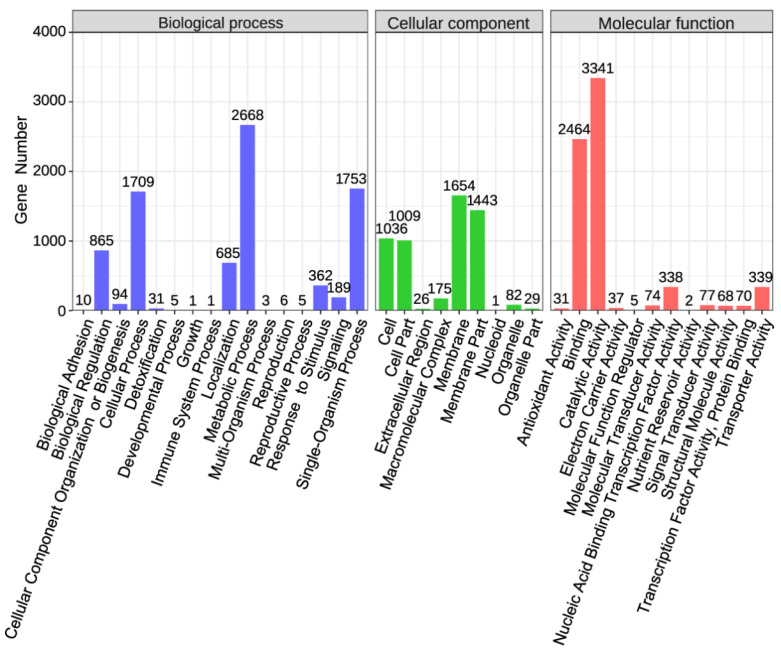
Gene Ontology (GO) enrichment analysis of the Qhu-G9 genome. The bar chart presents the functional categorization of predicted genes based on Gene Ontology (GO) terms, and they are divided into three major categories: Biological Process (blue), Cellular Component (green), and Molecular Function (red). Each bar represents the number of genes associated with a specific GO term.

The genes annotated in the GO database were classified into 1750 distinct terms (Figure 3 and Table S5). These terms were further categorized into cellular components, molecular functions, and biological processes, with a total of 5455, 6846, and 8387 genes annotated under each category, respectively. Within the molecular function category, genes associated with phosphate solubilization (GO: 0016791) and signal transducer activity (GO: 0016820) were identified. In terms of biological processes, genes involved in response to stimulus (GO: 0050896), colonization (GO: 0051179), detoxification (GO: 0098754), and bioadhesion (GO: 0005915) were annotated, with 362, 685, 31, and 10 genes, respectively. Notably, genes associated with catalytic activity, metabolic processes, and cellular processes were highly represented in the Qhu-G9 genome. These genes are predominantly involved in functions such as transmembrane transport, carbohydrate metabolism, amino acid metabolism, enzyme catalysis, and the synthesis of metabolic products, all of which are consistent with the plant growth-promoting and colonization capabilities of rhizosphere bacteria.

**Figure 4:**
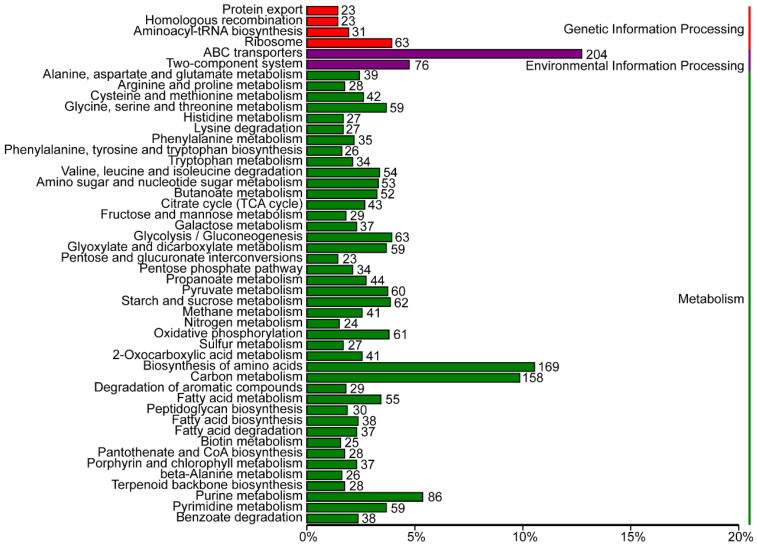
Kyoto Encyclopedia of Genes and Genomes (KEGG) metabolic pathway enrichment analysis of the Qhu-G9 genome. The genes are divided into three major categories: Genetic Information Processing (red), Environmental Information Processing (purple), and Metabolism (green). Each bar represents the number of genes associated with a specific KEGG pathway.

A total of 126 pathways were enriched in the KEGG pathway enrichment analysis, and these were categorized into three main groups (with 20 secondary pathways (Figure 4, Table S6)): Metabolism, Genetic Information Processing, and Environmental Information Processing. Among these, the pathway “ABC transporters” contained the largest number of genes, comprising 204 genes. This was followed by genes involved in “Biosynthesis of amino acids” (169), “carbohydrate metabolism” (158), and “purine metabolism” (86) (Figure 4 and Supplementary Table S5). Furthermore, 23 genes were annotated as participating in “homologous recombination,” a finding that aligns with the results from the unique gene cluster analysis. Additionally, within the metabolism category, genes such as indole-3-glycerol phosphate synthase (K01609), anthranilate synthase component I (K01657), and glutamine synthetase (K01915) were identified, indicating that Qhu-G9 possesses the potential to secrete IAA, solubilize phosphate, and synthesize glutathione, all of which probably contribute to its plant growth-promoting and stress-resilience traits.

### 2.3. Identification of Plant Growth-Promoting Genes in Qhu-G9

Through whole-genome sequencing, a series of plant growth-promoting genes were identified within the Qhu-G9 genome (Table S7). These included genes were found to be associated with environmental adaptation and regulation, such as the sensor histidine kinase genes *rcsC*, *dcuS*, *desK*, *pdtaS*, *tcrY*, and *dosT*. Genes involved in plant hormone IAA biosynthesis, including *trpS*, *trpD*, *trpE*, *iaaM*, and *trpG*, were also found. Additionally, a gene encoding ACC deaminase (*acdS*) and genes related to phosphate solubilization, such as *pstP* and *phnF*, were identified. Moreover, a cluster of genes related to the synthesis of glutamine (Gln) was found in the Qhu-G9 genome. Annotation of these genes, including *glnA*, *glnD*, *glnH*, *glnM*, and *glnQ*, suggests a role in nitrogen fixation, with these genes being responsible for the biosynthesis of Gln and are linked to nitrogenase activity. Furthermore, previous analyses of the secondary metabolite synthesis potential of Qhu-G9 revealed the presence of four gene clusters involved in the biosynthesis of siderophores, as well as genes encoding ferrous iron uptake proteins, including *efeU*, *efeN*, and *efeO*. Taken together, these findings strongly suggest that Qhu-G9 harbors a variety of plant growth-promoting traits, potentially enhancing plant growth and development through mechanisms such as nitrogen fixation, hormone production, and nutrient solubilization.

### 2.4. Evaluation of Plant Growth-Promoting Traits in Qhu-G9

**Figure 5:**
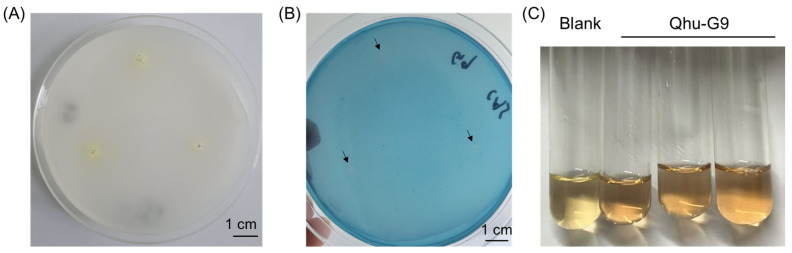
Evaluation of the growth-promoting properties of strain Qhu-G9. Phosphorus solubilization (**A**), siderophore production (**B**), and IAA synthesis assays (**C**).

Strain Qhu-G9 exhibited the ability to form clear halos on both Chrome Azurol S (CAS) and Pikovskaya (PKO) agar media, indicating its capacity to produce siderophores and solubilize phosphate (Figure 5A,B). Quantitative assessment of IAA production revealed that Qhu-G9 is capable of synthesizing and secreting IAA at a concentration of 4.37 μg·mL^−1^ (Figure 5C). Additionally, measurement of the ACC deaminase activity demonstrated that Qhu-G9 possesses high enzymatic activity, reaching 6.94 U·mg^−1^. The strain also displayed notable EPS production, with a concentration of 5.15 mg·mL^−1^, indicating a strong capacity for EPS synthesis. Collectively, these phenotypic characteristics suggest that Qhu-G9 harbors multiple rhizosphere–probiotic traits that can promote plant growth. These findings are in concordance with the genomic analyses, further supporting its potential as a plant-beneficial microbe.

### 2.5. Effect of Qhu-G9 on A. sativa Growth Promotion and Salt Stress Tolerance

**Figure 6:**
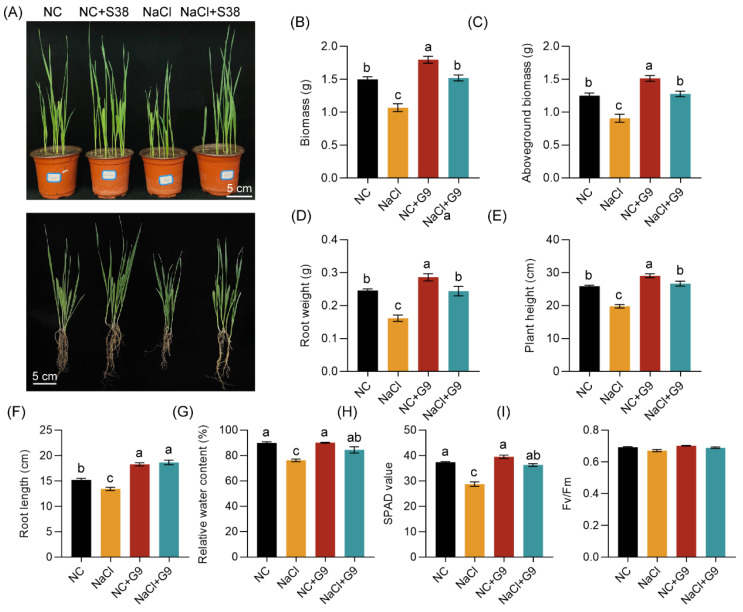
Effects of Qhu-G9 inoculation on the growth phenotype and physiological parameters of *A. sativa* seedlings. (**A**) Growth phenotypes of *A. sativa* seedlings with or without Qhu-G9 inoculation under normal and salt stress (200 mM NaCl) conditions. (**B**–**D**) Total biomass, shoot biomass, and root biomass of inoculated and uninoculated seedlings, respectively; each treatment included three replicates, with three seedlings per replicate. (**E**,**F**) Plant height and root length (n = 10). (**G**–**I**) Relative water content, chlorophyll content (SPAD value), and quantum efficiency of photosystem II (n = 3). NC, normal condition; NaCl, 200 mM NaCl treatment; NC + G9, Qhu-G9 inoculation under normal conditions; and NaCl + G9, Qhu-G9 inoculation under salt stress. Error bars represent standard deviations (SD). Significant differences among groups were determined by one-way ANOVA followed by Tukey’s test at *p* < 0.05, and they are indicated by different letters.

To further evaluate whether the Streptomyces strain Qhu-G9 could promote plant growth and enhance salt stress tolerance, we investigated its effects on the growth of *A. sativa*, a Graminae species, under both normal and salt stress conditions (Figure 6). The results revealed that salt stress severely inhibits the growth of *A. sativa* seedlings (Figure 6A). Compared to the NC group, the total biomass, shoot biomass, root weight, and plant height were significantly reduced by 28.5%, 27.5%, 34.2%, and 23.6%, respectively. However, inoculation with Qhu-G9 significantly alleviated these effects, increasing the total biomass, shoot biomass, root weight, and plant height by 20.0%, 20.8%, 16.4%, and 12.2%, respectively (Figure 6B–E). When compared to the NaCl-only stress group, *A. sativa* seedlings inoculated with Qhu-G9 (NaCl + G9) exhibited substantial improvements in total biomass, shoot biomass, root weight, and plant height, which increased by 42.1%, 40.9%, 50.9%, and 34.8%, respectively, with no significant difference from the NC group (Figure 6B–E).

Interestingly, salt stress significantly inhibited root development, reducing root length by 11.7% (Figure 6F). In contrast, inoculation with Qhu-G9 significantly enhanced root length under both normal and salt stress conditions, increasing by 20.0% and 38.7%, respectively, and it surpassed the NC group (Figure 6F). Furthermore, the relative water content and chlorophyll content in *A. sativa* seedlings displayed similar trends across different treatments (Figure 6G,H). Under salt stress conditions, both the relative water content and chlorophyll content were significantly reduced by 15.2% and 23.1%, respectively. However, in the NaCl + G9 group, the relative water content and chlorophyll content increased by 10.8% and 26.2%, respectively, compared to the NaCl-only stress group, with no significant difference from the NC group found (Figure 6G,H). Additionally, the Fv/Fm in *A. sativa* seedlings did not exhibit any statistical differences across the treatments (Figure 6I). These findings indicate that Streptomyces Qhu-G9 not only promotes the growth and development of *A. sativa*, but it also improves physiological parameters under salt stress, thereby enhancing the salt tolerance of *A. sativa* seedlings.

### 2.6. Qhu-G9 Promotes Growth and Salt Tolerance in O. viciifolia

**Figure 7:**
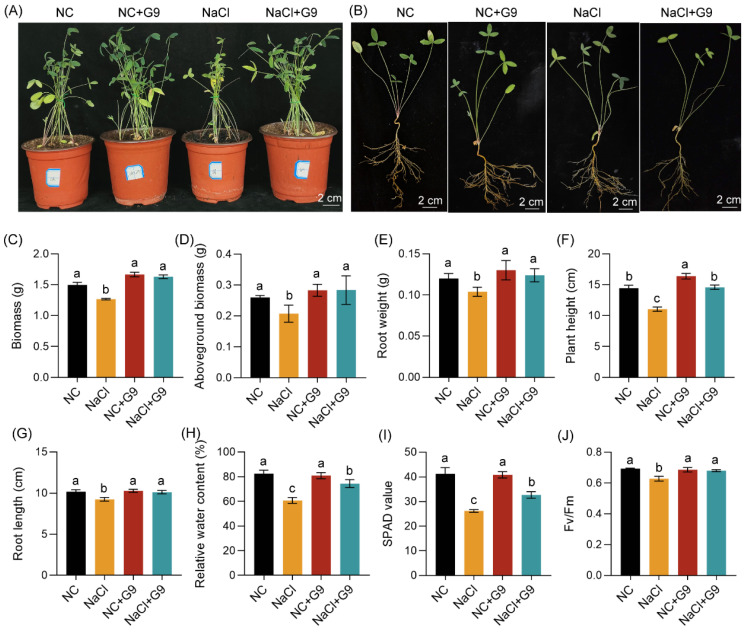
Effects of Qhu-G9 inoculation on the growth phenotype and physiological parameters of sainfoin seedlings. (**A**,**B**) Growth phenotypes of sainfoin seedlings with or without Qhu-G9 inoculation under normal and salt stress (200 mM NaCl) conditions. (**C**–**E**) Total biomass, shoot biomass, and root biomass of inoculated and uninoculated seedlings, respectively; each treatment included three replicates, with three seedlings per replicate. (**F**,**G**) Plant height and root length (n = 10). (**H**–**J**) Relative water content, chlorophyll content (SPAD value), and quantum efficiency of photosystem II (PSII) (n = 3). NC, normal condition; NaCl, 200 mM NaCl treatment; NC + G9, Qhu-G9 inoculation under normal conditions; and NaCl + G9, Qhu-G9 inoculation under salt stress. Error bars represent standard deviations (SD). Significant differences among groups were determined by one-way ANOVA followed by Tukey’s test at *p* < 0.05, and they are indicated by different letters.

To assess whether Qhu-G9 also confers growth-promoting and salt-tolerance benefits to other leguminous forage crops, we investigated its effects on *O. viciifolia* under NaCl-induced salt stress. After 14 days of salt stress, the growth of non-inoculated seedlings was severely suppressed, exhibiting pronounced leaf wilting and chlorosis. In contrast, seedlings inoculated with Qhu-G9 showed notable recovery in phenotype (Figure 7A,B).

Under salt stress conditions, compared to the non-inoculated NC group, the total biomass, shoot biomass, and root weight of *O. viciifolia* seedlings were significantly reduced by 15.4%, 20.2%, and 13.5%, respectively (Figure 7C–E). However, in both the NC + G9 and NaCl + G9 groups, these growth indices were comparable to those of the NC group, showing no significant differences. Notably, in comparison to the NaCl-only treatment, the NaCl + G9 group exhibited increases in total biomass, shoot biomass, and root weight by 28.7%, 36.9%, and 19.3%, respectively (Figure 7C–E). Inoculation with Qhu-G9 also enhanced plant height (Figure 7F). Under normal conditions, the plant height of seedlings in the NC + G9 group was significantly higher than in the NC group, with a 13.7% increase. Under salt stress conditions, the NaCl + G9 seedlings displayed plant height comparable to the NC group, effectively offsetting the 23.4% reduction caused by salt stress and showing a 32.0% increase relative to the NaCl-only group (Figure 7F).

Salt stress significantly inhibited root elongation, with the root length being reduced by 9.1% compared to the NC group. However, inoculation with Qhu-G9 restored root length to levels similar to those in the NC group, resulting in a 9.5% increase under salt stress conditions (Figure 7G). Physiological measurements further supported these observations. Compared with the NC group, the relative water content, chlorophyll content, and Fv/Fm in the NaCl group were significantly reduced by 26.4%, 36.5%, and 9.3%, respectively. In the NC + G9 group, these parameters remained stable (Figure 7H–J). Although still lower than in the NC group, the seedlings in the NaCl + G9 group showed marked improvements over the NaCl group, with relative increases of 22.5%, 24.7%, and 8.3% in water content, chlorophyll content, and Fv/Fm, respectively (Figure 7H–J). These phenotypic and physiological enhancements suggest that Qhu-G9 can mitigate the detrimental effects of salt stress and promote the growth and development of *O. viciifolia* seedlings.

### 2.7. Biochemical Responses of O. viciifolia Seedlings Inoculated with Qhu-G9 Under Salt Stress

**Figure 8:**
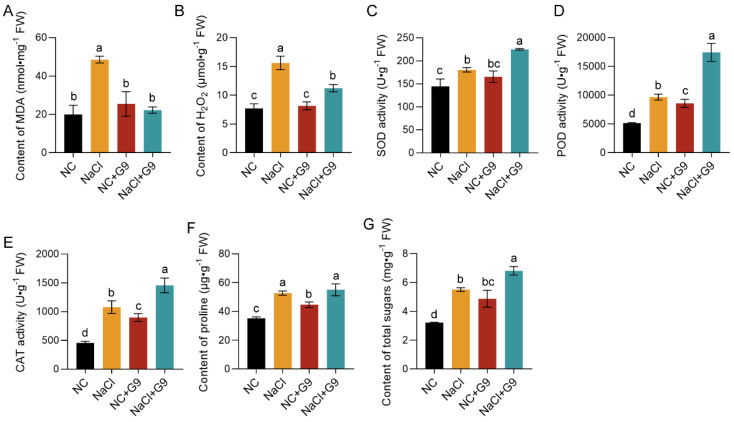
Effects of Qhu-G9 inoculation on the biochemical parameters of *O. viciifolia* seedlings. (**A**,**B**) Content of malondialdehyde (MDA) and hydrogen peroxide (H_2_O_2_) in sainfoin seedlings with or without Qhu-G9 inoculation under normal and salt stress (200 mM NaCl) conditions. (**C**–**E**) Activity of antioxidant enzymes superoxide dismutase (SOD), peroxidase (POD), and catalase (CAT) in inoculated and uninoculated seedlings. (**F**,**G**) Content of osmotic regulators proline and soluble sugars in inoculated and uninoculated sainfoin seedlings. All biochemical measurements were performed with three biological replicates. NC, normal condition; NaCl, 200 mM NaCl treatment; NC + G9, Qhu-G9 inoculation under normal conditions; and NaCl + G9, Qhu-G9 inoculation under salt stress. Error bars represent standard deviations (SD). Significant differences among groups were determined by one-way ANOVA followed by Tukey’s test at *p* < 0.05, and they are indicated by different letters.

To further explore the biochemical responses of *O. viciifolia* seedlings inoculated with Qhu-G9 under salt stress conditions, we analyzed various biochemical markers from different treatment groups. Under NaCl-induced salt stress, the contents of malondialdehyde (MDA) and hydrogen peroxide (H_2_O_2_) in *O. viciifolia* seedlings were significantly elevated, with increases of 143.1% and 102.7%, respectively, compared to the NC group (Figure 8A,B). These results suggest that salt stress induces severe oxidative stress in the seedlings. In contrast, inoculation with Qhu-G9 had a mitigating effect (Figure 8A,B). No significant changes in peroxide levels were observed in the NC + G9 group, while, in the NaCl + G9 group, the MDA and H_2_O_2_ levels decreased by 54.3% and 28.1%, respectively, with MDA returning to levels similar to those of the NC group (Figure 8A,B). This suggests that Qhu-G9 can alleviate the oxidative damage caused by salt stress.

Regarding antioxidant enzyme activity, Qhu-G9 inoculation significantly enhanced the activity of peroxidase (POD) and catalase (CAT) in *O. viciifolia* seedlings compared to the NC group, though superoxide dismutase (SOD) activity remained unchanged (Figure 8C–E). Under salt stress conditions, SOD, POD, and CAT activities were significantly elevated by 24.8%, 87.8%, and 136.2%, respectively, compared to the NC group (Figure 8C–E). Inoculation with Qhu-G9 further increased the activity of these enzymes under salt stress conditions. Specifically, in the NaCl + G9 group, the activities of SOD, POD, and CAT were increased by 24.7%, 80.1%, and 35.2% (Figure 8C–E), respectively, compared to the NaCl-only stress group, which likely contributed to enhanced peroxide scavenging ability. In terms of osmotic regulation, both salt stress and inoculation with Qhu-G9 induced the accumulation of osmotic regulators, including proline and soluble sugars, in the seedlings (Figure 8F,G). Compared to the NaCl treatment group, the proline content in the NaCl + G9 group did not significantly differ; however, the inoculated seedlings showed a marked increase in soluble sugar accumulation, with levels 23.5% higher than those in the NaCl-only group (Figure 8F,G). Taken together, these results suggest that Qhu-G9 enhances the antioxidant enzyme activities, reduces peroxide levels, and promotes the accumulation of osmotic regulators in *O. viciifolia* seedlings, thereby improving plant growth and enhancing salt stress tolerance.

## 3. Discussion

The use of PGPR is considered a key strategy for sustainable agriculture, as its successful application can reduce or even eliminate the need for pesticides and/or fertilizers without compromising crop yields [11]. Consequently, the discovery of salt-tolerant PGPR is of paramount importance in this context. In this study, through a comprehensive analysis—including whole-genome-based phylogenetic assessment, identification of growth-promoting genes, and evaluation of growth enhancement in *A. sativa* and *O. viciifolia* under salt stress conditions—we further validated the taxonomic position of Qhu-G9 and its potential in promoting plant growth under saline conditions.

The TYGS and UBGC2 methods are widely used for microbial taxonomic classification based on whole-genome sequencing [35,36], and both phylogenetic analyses placed Qhu-G9 as an independent branch distinct from other closely related strains (Figure 1 and Figure S1). This supports our earlier findings that Qhu-G9 is a potential new species of *Streptomyces*. Comparative genomic analysis with related strains revealed a significant number of genes associated with drought and salinity adaptation, such as DNA repair genes (*DnaC*, *SbcC*, and *XerD*), energy generation and conversion genes (*GlcD* and *SacC*), and carbohydrate transport and metabolism genes (*AmyA*, *UgpE*, and *PelB*) (Table S1). These genes may play crucial roles in the bacterium’s ability to adapt to extreme environmental conditions, further enhancing its potential as a bio-inoculant for saline environments.

Furthermore, the genome of Qhu-G9 harbors a significant number of plant growth-promoting genes (Table S7), including those involved in the synthesis of plant hormones, like IAA, and phosphate solubilization. Growth-promoting assays further confirmed that Qhu-G9 exhibits IAA production and phosphate-solubilizing capabilities (Figure 5A,C), which are commonly associated with beneficial microorganisms that promote plant growth [37,38]. Previous studies have also revealed that Qhu-G9′s genome contains biosynthetic gene clusters for iron siderophores, including Grincamycin and Desferrioxamine [34], as well as genes encoding iron uptake proteins, such as *efeU*, *efeN*, and *efeO*, suggesting the bacterium’s potential to produce iron carriers and facilitate the transport of iron–siderophore complexes [39]. Iron siderophore production was further confirmed in subsequent experiments (Figure 5B), indicating that Qhu-G9 shares this trait with other *Streptomyces* species, which are known for their widespread capacity to produce siderophores [40]. A similar finding was observed with the soybean endophytic bacterium *Leclercia adecarboxylata* (LSE-1), which produces siderophores that enhance soybean nodule formation and growth [41].

Additionally, Qhu-G9′s genome contains genes related to the synthesis of Gln, a key amino acid in plant nitrogen assimilation (Table S7). These genes suggest that Qhu-G9 may convert nitrate from the environment into Gln via the nitrate transport system, thus enhancing nitrogenase activity and providing plants with a source of nitrogen in the form of ammonia, which is readily available for plant growth [42]. The nitrogen fixation and nitrification activities of Qhu-G9, thus, contribute significantly to the nitrogen supply for host plants. Furthermore, Qhu-G9 was found to exhibit a robust ability to produce extracellular EPSs, which, along with the biofilm they form, help regulate the nutrient and water flow around plant roots [43]. These EPSs also prevent excessive Na^+^ absorption by plants, thereby mitigating the ion toxicity induced by salt stress [20]. Notably, numerous studies have indicated or implied that the ACC deaminase activity present in PGPR is a major mechanism by which these bacteria promote plant growth [44]. Moderate salt stress in plants induces growth inhibition and senescence, typically due to the production of low levels of ethylene. PGPR can alleviate this stress by secreting ACC deaminase, which prevents the production of ethylene, thereby safeguarding plants from the detrimental effects of salt stress [45]. Wang et al. isolated 13 PGPR strains with ACC deaminase activity from saline–alkaline soils in Xinjiang, China. Among them, three *Bacillus* strains significantly increased the fresh weight, dry weight, root length, and plant height of *Capsicum annuum* L. under salt stress conditions [46]; one of these strains, WU-9, enhanced proline levels and the antioxidant activity that help plants combat salt-induced toxicity [47].

The pot inoculation experiments further confirmed the plant growth-promoting potential of Qhu-G9. Under normal conditions, this *Streptomyces* sp. not only enhanced the biomass of *A. sativa* seedlings, including both above-ground and below-ground components, but also significantly increased plant height and root length (Figure 6), which are critical traits for forage productivity. However, under normal conditions, Qhu-G9 had a species-specific effect as it only influenced the plant height of *O. viciifolia* seedlings, while other physiological parameters were not significantly affected (Figure 7). This suggests that the growth-promoting effect of Qhu-G9 varies with plant species under non-stressed conditions. Nevertheless, Qhu-G9 was able to improve various physiological traits, such as biomass, plant height, root length, relative water content, and chlorophyll content, of both the *A. sativa* and *O. viciifolia* seedlings under salt stress conditions, restoring these traits to the levels observed under normal conditions (Figure 6 and Figure 7). This is consistent with findings from other studies, where *Streptomyces* strains (CLV97 and CLV179) isolated from the rhizosphere of *Brachiaria* sp. significantly promote the root and leaf growth in maize plants and alleviate the negative impacts of salinity on plant growth [48], suggesting that Qhu-G9 has broad potential to enhance plant salt tolerance.

Moreover, biochemical analysis revealed that Qhu-G9 mitigates the salt stress-induced damage in *O. viciifolia* by modulating the levels of oxidative stress markers, such as MDA and H_2_O_2_ [49]; enhancing antioxidant enzyme activity; and promoting the accumulation of osmotic regulators (Figure 8). In both the inoculated and non-inoculated *O. viciifolia* seedlings, MDA and H_2_O_2_ levels were significantly lower under salt stress conditions compared to the stressed control group, indicating that Qhu-G9 did not harm the plants but instead alleviated the oxidative stress caused by salt. This effect was likely linked to the significant increase in antioxidant enzyme activities induced by Qhu-G9, which are crucial for scavenging the reactive oxygen species (ROS) generated during salt stress [50]. Additionally, salt stress typically induces the accumulation of osmotic regulators to mitigate disturbances in osmotic potential [51]. In this study, both inoculated and non-inoculated *O. viciifolia* seedlings under salt stress conditions showed significantly higher levels of proline and soluble sugars compared to the normal control group. Moreover, inoculation with Qhu-G9 led to an even greater accumulation of these osmotic regulators, particularly soluble sugars, which may be related to Qhu-G9′s strong ability to produce extracellular EPSs. This suggests that Qhu-G9 plays a significant role in enhancing the synthesis and accumulation of soluble sugars in *O. viciifolia* seedlings under salt stress conditions, potentially contributing to the plant’s improved tolerance to salinity.

**Figure 9:**
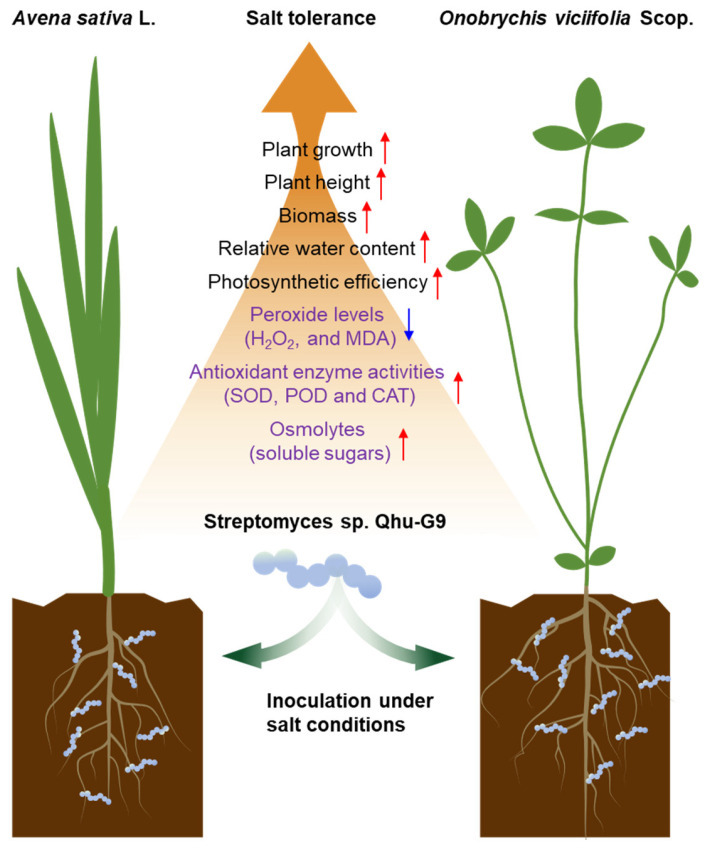
Schematic representation of the beneficial effects of Qhu-G9 on *A. sativa* and *O. viciifolia* under salt stress conditions. Red arrows indicate traits that are positively regulated upon Qhu-G9 inoculation, while blue arrows denote reduced levels. Biochemical parameters shown in purple—including peroxide levels, antioxidant enzyme activities, and osmolyte accumulation—were specifically measured in *O. viciifolia* seedlings.

Collectively, Qhu-G9 enhanced the salt stress tolerance of *A. sativa* and *O. viciifolia* by improving key physiological parameters, such as plant height, root length, biomass, relative water content, and photosynthetic efficiency. Furthermore, biochemical mechanisms underlying the improved stress resilience in *O. viciifolia* seedlings revealed that Qhu-G9 enhances antioxidant enzyme activities, promotes osmolyte accumulation (soluble sugars), and reduces peroxide levels, thereby strengthening the plant’s resistance to salinity (Figure 9). This study provides preliminary insights into the growth-promoting effects of Qhu-G9 and its physiological and biochemical regulatory mechanisms in two forage crops under salt stress conditions. In the future, incorporating transcriptomic, metabolomic, and microbiome analyses will further elucidate the molecular mechanisms through which Qhu-G9 enhances plant salt tolerance.

## 4. Materials and Methods

### 4.1. Microbial and Plant Materials

The strain Qhu-G9 used in this study was previously isolated from soil samples collected from a drought-prone habitat in Haixi Prefecture, Qinghai–Tibet Plateau (N37°19.8551′, E96°41.4545′). This strain was identified as a novel species of *Streptomyces* through a combination of multiple taxonomic methods. It is preserved at −80 °C in 20% glycerol at the College of Eco-Environmental Engineering, Qinghai University. Furthermore, this strain has been deposited in the China General Microbiological Culture Collection Center (CGMCC) under accession number CGMCC 4.8024, as well as in the Japan Collection of Microorganisms (JCM) under accession number JCM 37163. For the pot experiments, *A. sativa* and *O. viciifolia* were selected as the test plants. The experimental soil was a mixture of sand, peat, and vermiculite in a 1:1:1 ratio.

### 4.2. Whole-Genome Phylogenetic Analysis and Comparative Genomic Analysis

The sequenced genome was previously deposited in the GenBank database under accession number GCA027107535.1. For comprehensive whole-genome-based taxonomic analysis, the genome sequence data were subsequently submitted to the Type (Strain) Genome Server (TYGS), a freely accessible bioinformatics platform (https://tygs.dsmz.de, available at 18 April 2005). Pairwise comparisons among the genome set were conducted using the Genome BLAST Distance Phylogeny (GBDP) approach. Intergenomic distances were calculated using the ‘trimming’ algorithm with distance formula *d*5 [52]. In addition, a phylogenetic tree based on 81 conserved core genes was constructed using UBCG2 software following previously established protocols [53]. The average nucleotide identity based on ANIb was calculated using JSpecies to assess genomic similarity [54].

To further validate the taxonomic position and genomic distinctiveness of strain Qhu-G9 in comparison to related taxa, a comparative genomic analysis was performed using anvi’o v8.0 [55]. This analysis included comparisons with seven closely related species: *S. plumbiresistens* JCM 16924^T^ (GCA_039537125.1), *S. pseudovenezuelae* DSM 40212^T^ (GCA_001896135.2), *S. umbrinus* JCM 4958 (GCA_014656275.1), *S. phaeochromogenes* NRRL B-1248 (GCA_001418655.1), *S. liliifuscus* ZYC-3^T^ (GCA_016598615.1), *S. humidus* JCM 4386^T^ (GCA_014649655.1), and *S. asoensis* NBRC 13813^T^ (GCA_016860545.1).

### 4.3. Functional Annotation, Virulence Factor, and Genomic Islands Analysis

For functional annotation, protein-coding sequences were subjected to BLAST searches (E-value threshold: 1 × 10^−5^) against multiple public databases, including the Kyoto Encyclopedia of Genes and Genomes (KEGG), the Non-Redundant Protein Database (Nr), the Protein Families Database (Pfam), the Swiss-Prot protein sequence database, TrEMBL (Translated EMBL Nucleotide Sequence Database), and the Cluster of Orthologous Groups of proteins (eggNOG). Gene Ontology (GO) annotations were performed using Blast2GO. The predicted protein sequences were subjected to BLAST alignment against the core dataset of the virulence factor database (VFDB) to investigate the virulence genes in the strain [56]. In addition, genomic islands were predicted using IslandPath-DIMOB v0.2 and SIGI-HMM in the IslandViewer 4 platform [57].

### 4.4. Assays of the Plant Growth-Promoting Properties

To assess the phosphate solubilizing capability of the strain, Qhu-G9 was inoculated on a Pikovskaya (PKO) medium (glucose 10 g; Ca_3_(PO_4_)_2_ 5 g; MgCl_2_ 5 g; MgSO_4_·7H_2_O 0.25 g; KCl 0.2 g; (NH_4_)_2_SO_4_ 0.1 g; agar 15 g; and 1 L ddH_2_O) and incubated at 28 °C for 5 days. The presence of a clear zone around the colonies indicated phosphate solubilization activity. If no clear zone was observed, the strain was considered negative for phosphate solubilization [58]. The ability to produce siderophores was tested by inoculating Qhu-G9 on a Chrome Azurol S (CAS) agar medium (CAS 0.0605 g; Hexadecy-ltrimethyl-ammonium bromide 0.0729 g; FeCl_3_·6H_2_O 0.002645 g; NaH_2_(PO_4_)_2_·2H_2_O 0.29525 g; Na_2_H (PO_4_)_2_·12H_2_O 1.12135 g; NH_4_Cl 0.125 g; KH_2_(PO_4_)2 0.0375 g; NaCl 0.0625 g; agar 9 g; and 1 L ddH_2_O); after 5 days of incubation at 28 °C, the formation of a transparent halo around the colonies indicated siderophore production. IAA content determination: the strain was inoculated into a liquid medium containing tryptophan (100 mg·L^−1^) and cultured under shaking conditions at 28 °C for 48 h. After centrifugation, 2 mL of the supernatant was mixed with 50 μL of 83% orthophosphoric acid and 4 mL of Salkowski reagent. The appearance of a pink color indicated the production of IAA. A standard IAA solution underwent the same treatment to generate a standard curve, and the IAA concentration in the sample was determined by measuring the optical density (OD) at 530 nm, applying the linear equation derived from the standard curve. EPS content determination: the strain was first cultured in a Trypticase Soy Broth (TSB) medium for 2 days, after which equal volumes of deionized water were added, followed by centrifugation to collect the supernatant. Then, 2 volumes of 95% ethanol were added to the supernatant, mixed well, and centrifuged to collect the precipitate, which represented the crude polysaccharides. The phenol-sulfuric acid method was used to construct a glucose standard curve. A 1.0 mL sample was mixed with 1.0 mL of distilled water. Then, 1.0 mL of 6% phenol was added, followed by 5.0 mL of concentrated sulfuric acid. The mixture was thoroughly mixed and left to stand for 30 min. The absorbance was measured at 490 nm, and the polysaccharide concentration was calculated from the standard curve. The activity of ACC deaminase was measured following the methodology we previously reported [58].

### 4.5. Preparation of Bacterial Suspension

Qhu-G9 was streaked onto a Tryptic Soy Agar (TSA) medium and incubated at 28 °C for 96 h. Single colonies were selected and inoculated into a TSB medium, where they were cultured at 28 °C and 180 rpm for 72 h. Cells were harvested by centrifugation at 6000× *g* for 5 min. Next, they were resuspended in sterile deionized water, and the bacterial suspension was then adjusted to 1.0 × 10^8^ cfu/mL for use in inoculation experiments.

### 4.6. Pot Experiments and Salt Stress Treatment

Healthy, plump seeds of *A. sativa* and *O. viciifolia* were surface-sterilized by immersion in 2% sodium hypochlorite for 5 min, followed by 45 s of exposure to 75% ethanol, and they then were washed 5 times with sterile deionized water. The seeds were then sown in round pots (diameter 10 cm, height 9 cm) containing a mixture of peat, vermiculite, and sand (1:1:1, *v*/*v*). Five *O. viciifolia* seeds and seven *A. sativa* seeds were sown per pot. The plants were watered every three days, and the growth conditions were set to a 16 h light/8 h dark photoperiod with a light intensity of 8000 lux at a temperature of 22–25 °C. The pot cultivation and inoculation experiments included four treatment groups: plants grown under normal conditions (NC), plants inoculated with Qhu-G9 under normal conditions (NC + G9), plants subjected to 200 mM NaCl stress (NaCl), and plants inoculated with Qhu-G9 under salt stress (NaCl + G9). After seed germination, *A. sativa* and *O. viciifolia* medic were grown for 7 and 24 days, respectively. The NC + G9 and NaCl + G9 groups were irrigated with 30 mL of bacterial suspension at the root zone, while NC and NaCl groups received the same volume of sterile deionized water. A second inoculation was performed one day later. Following the two inoculations, the NaCl and NaCl + G9 groups were irrigated daily with 50 mL of 200 mM NaCl solution for two consecutive days. After 10 and 14 days of salt stress, the plant phenotypes were, respectively, photographed and observed, and the plant tissues were then collected for subsequent measurements of the physiological and biochemical parameters.

### 4.7. Plant Physiological and Biochemical Assays

Plant height and root length in different treatment groups were measured using a tape measure (n = 10). The total plant biomass, shoot biomass, and root weight were determined using an analytical balance with a precision of 0.0001 g. Three seedlings of similar size were selected as a replicate, and each measurement was performed in triplicate. For relative water content, 0.2 g of fully expanded leaves from the same plant were sampled, flash frozen in liquid nitrogen, and then dried in an oven at 105 °C for 15 min, followed by drying at 80 °C to a constant weight. The fresh weight (FW), turgid weight (TW), and dry weight (DW) were recorded, and the relative water content was calculated as follows: relative water content (%) = (FW − DW)/(TW − DW) × 100, where FW, TW, and DW represent the fresh weight, turgid weight, and dry weight, respectively. The quantum efficiency of photosystem II (Fv/Fm) was measured using a chlorophyll fluorometer (FluorPen FP110, PSI, Praha, Czech Republic), and the leaf chlorophyll content was quantified using a chlorophyll meter (SPAD 502 Plus, Konicaminolta, Tokyo, Japan). For biochemical assays, fresh leaf samples (0.1 g) were collected from *A. sativa* and *O. viciifolia* seedlings. These were then ground in liquid nitrogen and analyzed using commercial kits (purchased from Keming Biotechnology Co., Ltd., Suzhou, China) to determine the activities of superoxide dismutase (SOD), peroxidase (POD), and catalase (CAT), as well as the concentrations of soluble sugars, proline, malondialdehyde (MDA), and hydrogen peroxide (H_2_O_2_). Each experiment included three biological replicates.

### 4.8. Statistical Analysis

Data were analyzed using one-way analysis of variance (ANOVA) in SPSS 26.0. Prior to performing ANOVA, the assumptions of normality and homogeneity of variance were assessed. Normality of the data distribution was evaluated using the Shapiro–Wilk test, while Levene’s test was applied to assess the homogeneity of variances. Upon validation of these assumptions, Tukey’s post hoc test was used for multiple comparisons, with *p* < 0.05 denoting significance. Graphical representations were generated using GraphPad Prism 8.0.

## 5. Conclusions

In summary, *Streptomyces* Qhu-G9 exhibits multifaceted plant growth-promoting traits and plays a crucial role in enhancing the salt stress tolerance in Graminae or Leguminous forage crops. Through genomic and physiological analyses, we demonstrated that Qhu-G9 can significantly improve plant biomass, antioxidant enzyme activity, and osmotic adjustment, while reducing oxidative damage under saline conditions. These benefits are likely mediated by its ability to produce IAA, solubilize phosphate, secrete siderophores, and modulate stress-related biochemical pathways. The promising results from both controlled and stress conditions support Qhu-G9 as a viable candidate for bio-inoculant development aimed at improving crop performance in salt-affected soils. Looking forward, further investigation into the molecular mechanisms governing plant–microbe interactions will deepen our understanding of Qhu-G9′s functional roles. Additionally, field-scale trials across different soil types and climatic conditions will be essential to validate its effectiveness and stability, paving the way for its application in sustainable agriculture.

## Figures and Tables

**Table 1:** Summary of the annotated gene features in the whole genome of strain Qhu-G9.

Public Database	Annotated Gene Number	100 bp ≤ Length < 300 bp	Length ≥ 300 bp
eggNOG_Annotation	6529	2759	3519
GO_Annotation	5824	2355	3200
KEGG_Annotation	2849	1018	1742
Nr_Annotation	8515	3850	4034
Pfam_Annotation	6792	2916	3643
Swissprot_Annotation	3964	1358	2514
TrEMBL_Annotation	3964	1358	2514
All_Annotated	8525	3852	4035

## Data Availability

The sequenced genome for this study has been deposited into the GenBank under accession number GCA027107535.1.

## Supplementary Materials

The following supporting information can be downloaded at: https://www.mdpi.com/article/10.3390/plants14142135/s1, Figure S1: Phylogenomic tree constructed with UBCG2 software using 81 core genes; Figure S2: Annotation results of the virulence genes in strain Qhu-G9; Figure S3: Predicted genomic islands in the Qhu-G9 genome; Table S1: Unique gene clusters identified in the genome of Qhu-G9; Table S2: Annotation of the virulence genes in the genome of Qhu-G9; Table S3: Predicted genomic island information in the Qhu-G9 genome; Table S4: Annotation of the coding genes in the Qhu-G9 genome in public databases; Table S5: GO enrichment analysis of the Qhu-G9 genome; Table S6: KEGG pathway enrichment analysis based on the whole-genome sequence of Qhu-G9; and Table S7: The plant growth-promoting genes identified in the Qhu-G9 genome.

## Abbreviations

ACC	1-Aminocyclopropane-1-carboxylate
ANIb	ANI algorithm using BLAST
ANOVA	One-way analysis of variance
CAT	Catalase
EPSs	Exopolysaccharides
GBDP	Genome BLAST Distance Phylogeny
H_2_O_2_	Hydrogen peroxide
IAA	Indole-3-acetic acid
MDA	Malondialdehyde
PGPR	Plant growth-promoting rhizobacterium
POD	Peroxidase
Fv/Fm	Quantum efficiency of photosystem II
SOD	Superoxide dismutase
TYGS	Type (Strain) Genome Server
UBCG2	Up-to-Date Bacterial Core Gene 2

## References

[1] Van Zelm E., Zhang Y., Testerink C. (2020). Salt Tolerance Mechanisms of Plants. Annu. Rev. Plant Biol..

[2] Shahid S.A., Zaman M., Heng L., Zaman M., Shahid S.A., Heng L. (2018). Soil Salinity: Historical Perspectives and a World Overview of the Problem. Guideline for Salinity Assessment, Mitigation and Adaptation Using Nuclear and Related Techniques.

[3] Wang X., Du L., Wang W., Zhang Z., Wu Y., Wang Y. (2023). Functional identification of ZDS gene in apple (*Malus halliana*) and demonstration of it’s role in improving saline-alkali stress tolerance. Physiol. Mol. Biol. Plants.

[4] Zhang T., Han J., Zhang H. (2023). Rapid saline-alkali sensitivity testing using hydrogel/gold nanoparticles-modified screen-printed electrodes. Sci. Total Environ..

[5] Durán P., Thiergart T., Garrido-Oter R., Agler M., Kemen E., Schulze-Lefert P., Hacquard S. (2018). Microbial Interkingdom Interactions in Roots Promote Arabidopsis Survival. Cell.

[6] Tkacz A., Poole P. (2015). Role of root microbiota in plant productivity. J. Exp. Bot..

[7] Krishnan K.S., Rangasamy A., Arunan Y.E., Dananjeyan B., Subramanium T., Saminathan V. (2025). Microbial inoculants—Fostering sustainability in groundnut production. Sci. Prog..

[8] Li J., Wang J., Liu H., Macdonald C.A., Singh B.K. (2023). Microbial inoculants with higher capacity to colonize soils improved wheat drought tolerance. Microb. Biotechnol..

[9] Narsing Rao M.P., Lohmaneeratana K., Bunyoo C., Thamchaipenet A. (2022). Actinobacteria-Plant Interactions in Alleviating Abiotic Stress. Plants.

[10] Al-Turki A., Murali M., Omar A.F., Rehan M., Sayyed R.Z. (2023). Recent advances in PGPR-mediated resilience toward interactive effects of drought and salt stress in plants. Front. Microbiol..

[11] He S., Li L., Lv M., Wang R., Wang L., Yu S., Gao Z., Li X. (2024). PGPR: Key to Enhancing Crop Productivity and Achieving Sustainable Agriculture. Curr. Microbiol..

[12] Giannelli G., Potestio S., Visioli G. (2023). The Contribution of PGPR in Salt Stress Tolerance in Crops: Unravelling the Molecular Mechanisms of Cross-Talk between Plant and Bacteria. Plants.

[13] Yin Z., Wang X., Hu Y., Zhang J., Li H., Cui Y., Zhao D., Dong X., Zhang X., Liu K. (2022). *Metabacillus dongyingensis* sp. nov. Is Represented by the Plant Growth-Promoting Bacterium BY2G20 Isolated from Saline-Alkaline Soil and Enhances the Growth of *Zea mays* L. under Salt Stress. mSystems.

[14] Shang X., Hui L., Jianlong Z., Hao Z., Cao C., Le H., Weimin Z., Yang L., Gao Y., Hou X. (2023). The application of plant growth-promoting rhizobacteria enhances the tolerance of tobacco seedling to salt stress. Ecotoxicol. Environ. Saf..

[15] Zhang H., Zhu J., Gong Z., Zhu J.K. (2022). Abiotic stress responses in plants. Nat. Rev. Genet..

[16] Mahanty T., Bhattacharjee S., Goswami M., Bhattacharyya P., Das B., Ghosh A., Tribedi P. (2017). Biofertilizers: A potential approach for sustainable agriculture development. Environ. Sci. Pollut. Res. Int..

[17] Ferreira C.M.H., Soares H., Soares E.V. (2019). Promising bacterial genera for agricultural practices: An insight on plant growth-promoting properties and microbial safety aspects. Sci. Total Environ..

[18] Li H., Lei P., Pang X., Li S., Xu H., Xu Z., Feng X. (2017). Enhanced tolerance to salt stress in canola (*Brassica napus* L.) seedlings inoculated with the halotolerant *Enterobacter cloacae* HSNJ4. Appl. Soil Ecol..

[19] Qurashi A.W., Sabri A.N. (2012). Bacterial exopolysaccharide and biofilm formation stimulate chickpea growth and soil aggregation under salt stress. Braz. J. Microbiol..

[20] Liu X., Yao T., Chai J., Han J. (2023). Adsorption of Sodium Ions by Exopolysaccharides from *Pseudomonas simiae* MHR6 and Its Improvement of Na^+^/K^+^ Homeostasis in Maize under Salt Stress. J. Agric. Food Chem..

[21] Ayaz M., Ali Q., Jiang Q., Wang R., Wang Z., Mu G., Khan S.A., Khan A.R., Manghwar H., Wu H. (2022). Salt Tolerant Bacillus Strains Improve Plant Growth Traits and Regulation of Phytohormones in Wheat under Salinity Stress. Plants.

[22] Shabaan M., Asghar H.N., Zahir Z.A., Zhang X., Sardar M.F., Li H. (2022). Salt-Tolerant PGPR Confer Salt Tolerance to Maize Through Enhanced Soil Biological Health, Enzymatic Activities, Nutrient Uptake and Antioxidant Defense. Front. Microbiol..

[23] Sultana S., Paul S.C., Parveen S., Alam S., Rahman N., Jannat B., Hoque S., Rahman M.T., Karim M.M. (2020). Isolation and identification of salt-tolerant plant-growth-promoting rhizobacteria and their application for rice cultivation under salt stress. Can. J. Microbiol..

[24] Etesami H., Maheshwari D.K. (2018). Use of plant growth promoting rhizobacteria (PGPRs) with multiple plant growth promoting traits in stress agriculture: Action mechanisms and future prospects. Ecotoxicol. Environ. Saf..

[25] Maurya N., Sharma A., Sundaram S. (2024). The Role of PGPB-Microalgae interaction in Alleviating Salt Stress in Plants. Curr. Microbiol..

[26] Pang F., Solanki M.K., Wang Z. (2022). *Streptomyces* can be an excellent plant growth manager. World J. Microbiol. Biotechnol..

[27] Akbari A., Gharanjik S., Koobaz P., Sadeghi A. (2020). Plant growth promoting *Streptomyces strains* are selectively interacting with the wheat cultivars especially in saline conditions. Heliyon.

[28] Lu L., Liu N., Fan Z., Liu M., Zhang X., Tian J., Yu Y., Lin H., Huang Y., Kong Z. (2024). A novel PGPR strain, *Streptomyces lasalocidi* JCM 3373(T), alleviates salt stress and shapes root architecture in soybean by secreting indole-3-carboxaldehyde. Plant Cell Environ..

[29] Gong Y., Chen L.-J., Pan S.-Y., Li X.-W., Xu M.-J., Zhang C.-M., Xing K., Qin S. (2020). Antifungal potential evaluation and alleviation of salt stress in tomato seedlings by a halotolerant plant growth-promoting actinomycete *Streptomyces* sp. KLBMP5084. Rhizosphere.

[30] Zhou X., Wang M., Yang L., Wang W., Zhang Y., Liu L., Chai J., Liu H., Zhao G. (2024). Comparative Physiological and Transcriptomic Analyses of Oat (*Avena sativa*) Seedlings under Salt Stress Reveal Salt Tolerance Mechanisms. Plants.

[31] Xu Z., Chen X., Lu X., Zhao B., Yang Y., Liu J. (2021). Integrative analysis of transcriptome and metabolome reveal mechanism of tolerance to salt stress in oat (*Avena sativa* L.). Plant Physiol. Biochem..

[32] Yin H., Zhou H., Wang W., Tran L.P., Zhang B. (2020). Transcriptome Analysis Reveals Potential Roles of Abscisic Acid and Polyphenols in Adaptation of *Onobrychis viciifolia* to Extreme Environmental Conditions in the Qinghai-Tibetan Plateau. Biomolecules.

[33] Kang K.K., Cho Y.G. (2022). Genetic Research and Plant Breeding. Genes.

[34] Xiang X., Yin H.X., Fan J.K., Deng J.W., Zhu Z.Y., Qiu Q.H., Liu Y.R., Zhang B.Y. (2023). Polyphasic identification and genome mining of secondary metabolites of a novel species *Streptomyces haixigobicum* sp. nov. Qhu-G9 isolated from the Gobi habitat of the Qinghai-Tibetan Plateau. Microbiol. China.

[35] Meier-Kolthoff J.P., Carbasse J.S., Peinado-Olarte R.L., Göker M. (2022). TYGS and LPSN: A database tandem for fast and reliable genome-based classification and nomenclature of prokaryotes. Nucleic Acids Res..

[36] Tambong J.T., Xu R., Fleitas M.C., Wang L., Hubbard K., Kutcher R. (2023). Phylogenomic Insights on the Xanthomonas translucens Complex, and Development of a TaqMan Real-Time Assay for Specific Detection of pv. translucens on Barley. Phytopathology.

[37] Pishchik V.N., Chizhevskaya E.P., Chebotar V.K., Mirskaya G.V., Khomyakov Y.V., Vertebny V.E., Kononchuk P.Y., Kudryavtcev D.V., Bortsova O.A., Lapenko N.G. (2024). PGPB Isolated from Drought-Tolerant Plants Help Wheat Plants to Overcome Osmotic Stress. Plants.

[38] Samayoa B.E., Shen F.T., Lai W.A., Chen W.C. (2020). Screening and Assessment of Potential Plant Growth-promoting Bacteria Associated with *Allium cepa* Linn. Microbes Environ..

[39] do Amaral F.P., Tuleski T.R., Pankievicz V.C.S., Melnyk R.A., Arkin A.P., Griffitts J., Tadra-Sfeir M.Z., Maltempi de Souza E., Deutschbauer A., Monteiro R.A. (2020). Diverse Bacterial Genes Modulate Plant Root Association by Beneficial Bacteria. mBio.

[40] Zang Z., Zhang C., Park K.J., Schwartz D.A., Podicheti R., Lennon J.T., Gerdt J.P. (2025). *Streptomyces* secretes a siderophore that sensitizes competitor bacteria to phage infection. Nat. Microbiol..

[41] Kumawat K.C., Sharma P., Singh I., Sirari A., Gill B.S. (2019). Co-existence of Leclercia adecarboxylata (LSE-1) and *Bradyrhizobium* sp. (LSBR-3) in nodule niche for multifaceted effects and profitability in soybean production. World J. Microbiol. Biotechnol..

[42] Nordlund S., Högbom M. (2013). ADP-ribosylation, a mechanism regulating nitrogenase activity. FEBS J..

[43] Limoli D.H., Jones C.J., Wozniak D.J. (2015). Bacterial Extracellular Polysaccharides in Biofilm Formation and Function. Microbiol. Spectr..

[44] Singh R.P., Ma Y., Shadan A. (2022). Perspective of ACC-deaminase producing bacteria in stress agriculture. J. Biotechnol..

[45] Glick B.R. (2014). Bacteria with ACC deaminase can promote plant growth and help to feed the world. Microbiol. Res..

[46] Wang W., Wu Z., He Y., Huang Y., Li X., Ye B.C. (2018). Plant growth promotion and alleviation of salinity stress in *Capsicum annuum* L. by *Bacillus* isolated from saline soil in Xinjiang. Ecotoxicol. Environ. Saf..

[47] Wang W., He Y., Wu Z., Li T., Xu X., Liu X. (2022). De novo transcriptome sequencing of *Capsicum frutescens* L. and comprehensive analysis of salt stress alleviating mechanism by *Bacillus atrophaeus* WU-9. Physiol. Plant.

[48] Nozari R.M., Ortolan F., Astarita L.V., Santarém E.R. (2021). *Streptomyces* spp. enhance vegetative growth of maize plants under saline stress. Braz. J. Microbiol..

[49] Kurniawan A., Chuang H.W. (2022). Rhizobacterial *Bacillus mycoides* functions in stimulating the antioxidant defence system and multiple phytohormone signalling pathways to regulate plant growth and stress tolerance. J. Appl. Microbiol..

[50] Wang G., Zhang L., Zhang S., Li B., Li J., Wang X., Zhang J., Guan C., Ji J. (2023). The combined use of a plant growth promoting *Bacillus* sp. strain and GABA promotes the growth of rice under salt stress by regulating antioxidant enzyme system, enhancing photosynthesis and improving soil enzyme activities. Microbiol. Res..

[51] Tian T., Wang J., Wang H., Cui J., Shi X., Song J., Li W., Zhong M., Qiu Y., Xu T. (2022). Nitrogen application alleviates salt stress by enhancing osmotic balance, ROS scavenging, and photosynthesis of rapeseed seedlings (*Brassica napus*). Plant Signal. Behav..

[52] Meier-Kolthoff J.P., Göker M. (2019). TYGS is an automated high-throughput platform for state-of-the-art genome-based taxonomy. Nat. Commun..

[53] Kim J., Na S.I., Kim D., Chun J. (2021). UBCG2: Up-to-date bacterial core genes and pipeline for phylogenomic analysis. J. Microbiol..

[54] Richter M., Rosselló-Móra R., Oliver Glöckner F., Peplies J. (2016). JSpeciesWS: A web server for prokaryotic species circumscription based on pairwise genome comparison. Bioinformatics.

[55] Eren A.M., Kiefl E., Shaiber A., Veseli I., Miller S.E., Schechter M.S., Fink I., Pan J.N., Yousef M., Fogarty E.C. (2021). Community-led, integrated, reproducible multi-omics with anvi’o. Nat. Microbiol..

[56] Zhou S., Liu B., Zheng D., Chen L., Yang J. (2025). VFDB 2025: An integrated resource for exploring anti-virulence compounds. Nucleic Acids Res..

[57] Bertelli C., Laird M.R., Williams K.P., Lau B.Y., Hoad G., Winsor G.L., Brinkman F.S.L. (2017). IslandViewer 4: Expanded prediction of genomic islands for larger-scale datasets. Nucleic Acids Res..

[58] Ma X., Zhang B., Xiang X., Li W., Li J., Li Y., Tran L.P., Yin H. (2024). Characterization of *Bacillus pacificus* G124 and Its Promoting Role in Plant Growth and Drought Tolerance. Plants.

