# Supplementary material for: Genome-Wide Insights into Streptomyces Novel Species Qhu-G9 and Its Potential for Enhancing Salt Tolerance and Growth in Avena sativa L. and Onobrychis viciifolia Scop
Source: Plants (Basel). 2025 Jul 10;14(14):2135. doi: 10.3390/plants14142135 (PMC12298133; doi:10.3390/plants14142135)
Supplement: Supplementary file 1 [file plants-14-02135-s001.zip › Supplementary Figures_Plants.pdf]

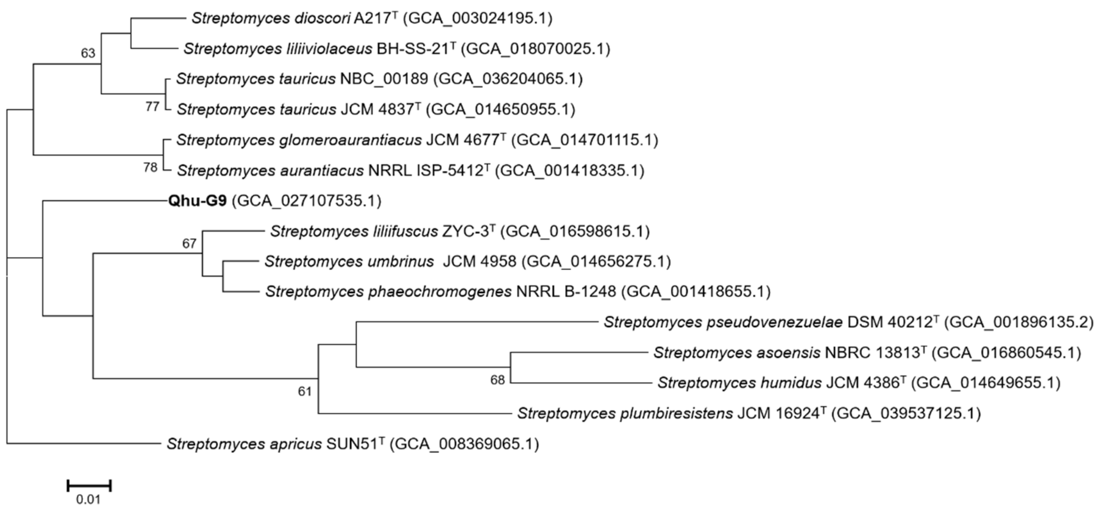

Figure S1 Phylogenomic tree constructed with UBCG2 software using 81 core genes. Bootstrap values greater than 50% are displayed at the nodes, indicating the level of support for each clade. The tree illustrates the evolutionary relationships among the strains, with branch lengths representing genetic distances.

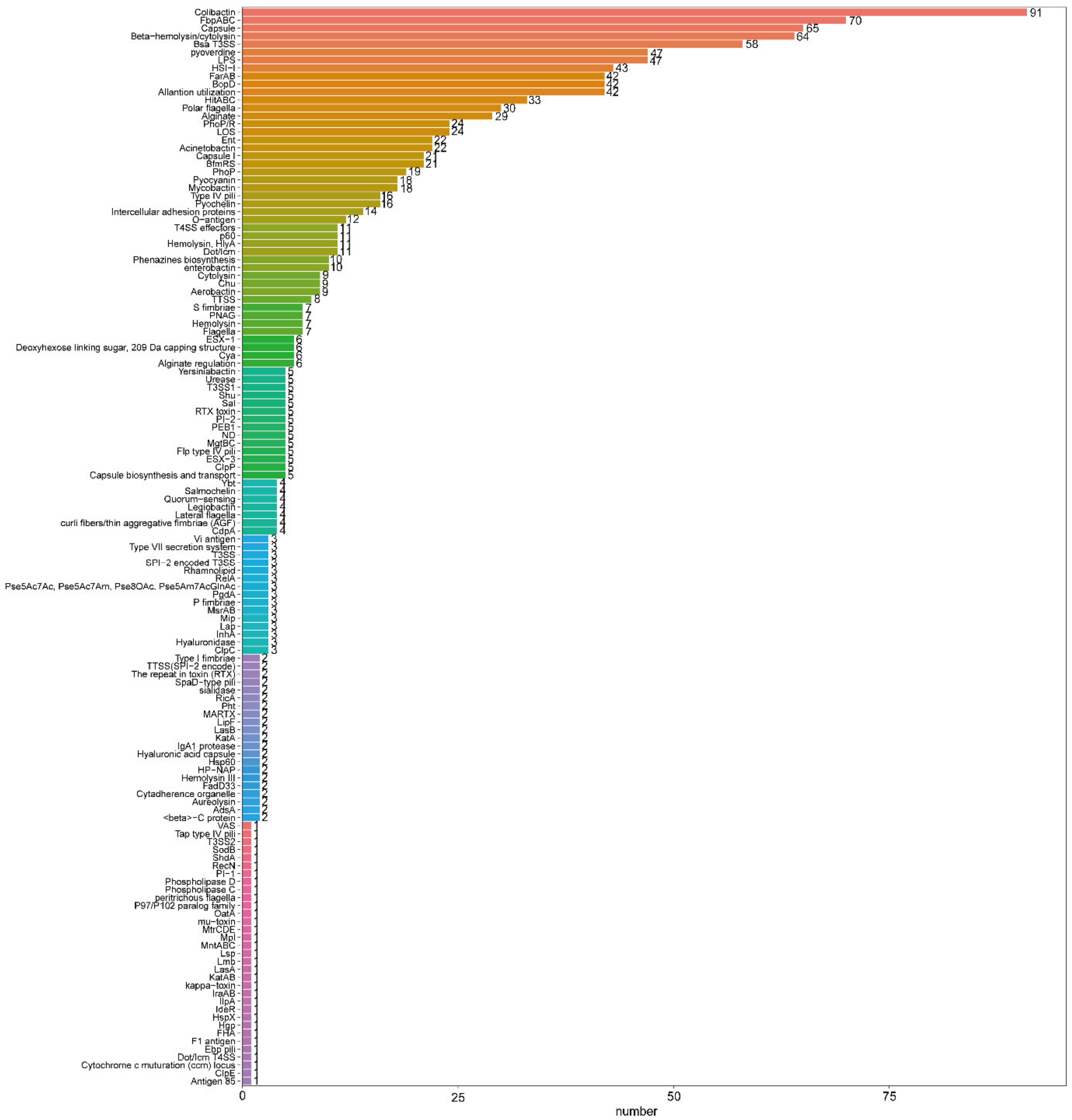

Figure S2 Annotation results of virulence genes in strain Qhu-G9.

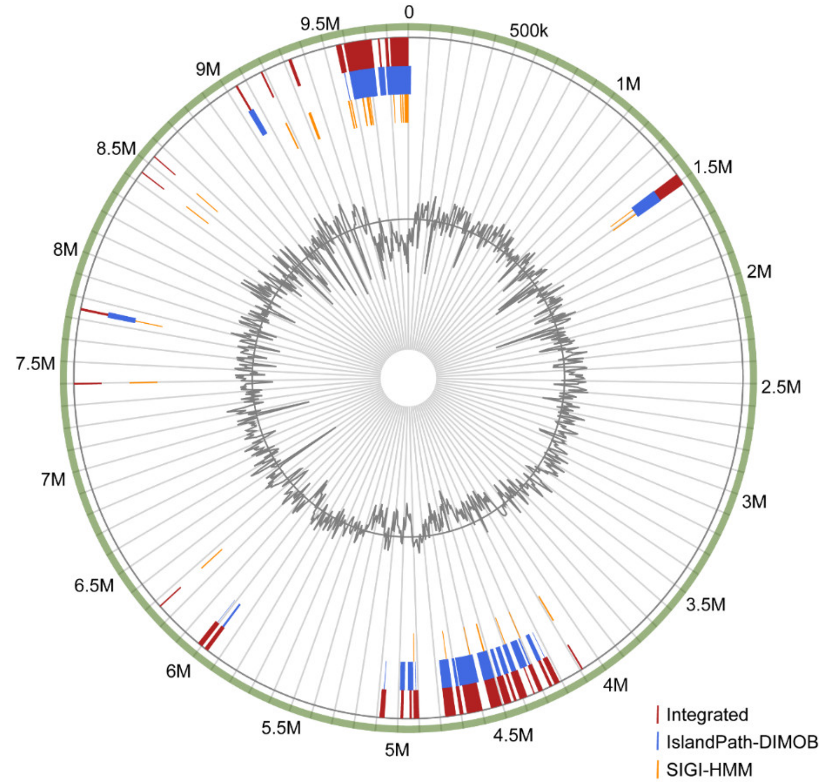

Figure S3 Predicted genomic islands in the Qhu-G9 genome.
